# Phytochemical Constituents, Antioxidant Activity, and Toxicity Assessment of the Aerial Part Extracts from the Infraspecific Taxa of *Matthiola fruticulosa* (*Brassicaceae*) Endemic to Sicily

**DOI:** 10.3390/molecules26144114

**Published:** 2021-07-06

**Authors:** Maria Fernanda Taviano, Emilia Cavò, Vivienne Spadaro, Francesco Maria Raimondo, Vincenzo Musolino, Francesco Cacciola, Yassine Oulad El Majdoub, Luigi Mondello, Concetta Condurso, Fabrizio Cincotta, Antonella Verzera, Natalizia Miceli

**Affiliations:** 1 Department of Chemical, Biological, Pharmaceutical and Environmental Sciences, University of Messina, Viale Palatucci, 98168 Messina, Italy; ecavo@unime.it (E.C.); youladelmajdoub@unime.it (Y.O.E.M.); lmondello@unime.it (L.M.); nmiceli@unime.it (N.M.); 2 Foundation “Prof. Antonio Imbesi”, University of Messina, Piazza Pugliatti 1, 98122 Messina, Italy; 3 Department STEBICEF/Section of Botany, Anthropology and Zoology, University of Palermo, Via Archirafi 38, 90123 Palermo, Italy; vivienne.spadaro@unipa.it; 4 PLANTA/Research, Documentation and Training Center, Via Serraglio Vecchio 28, 90123 Palermo, Italy; raimondo@centroplantapalermo.org; 5 Department of Health Sciences, University “Magna Graecia” of Catanzaro, V. Europa, Località Germaneto, 88100 Catanzaro, Italy; v.musolino@unicz.it; 6 Department of Biomedical, Dental, Morphological and Functional Imaging Sciences, University of Messina, Via Consolare Valeria, 98125 Messina, Italy; cacciolaf@unime.it; 7 Chromaleont s.r.l., c/o Department of Chemical, Biological, Pharmaceutical and Environmental Sciences, University of Messina, Viale Palatucci, 98168 Messina, Italy; 8 BeSep s.r.l., c/o Department of Chemical, Biological, Pharmaceutical and Environmental Sciences, University of Messina, Viale Palatucci, 98168 Messina, Italy; 9 Department of Sciences and Technologies for Human and Environment, University Campus Bio-Medico of Rome, Via Àlvaro del Portillo 21, 00128 Rome, Italy; 10 Department of Veterinary Sciences, University of Messina, Viale Palatucci, 98168 Messina, Italy; concetta.condurso@unime.it (C.C.); fabrizio.cincotta@unime.it (F.C.); antonella.verzera@unime.it (A.V.)

**Keywords:** native plants, Sicily, natural resource, *Matthiola fruticulosa*, chemical composition, biological activity

## Abstract

In a project designed to investigate the specific and infraspecific taxa of *Matthiola* endemic to Sicily (Italy) as new potential sources of bioactive compounds in this work, the infraspecific taxa of *Matthiola fruticulosa* were studied, namely, subsp. *fruticulosa* and subsp. *coronopifolia*. HPLC–PDA/ESI–MS and SPME–GC/MS analyses of hydroalcoholic extracts obtained from the aerial parts of the two subspecies led to the detection of 51 phenolics and 61 volatile components, highlighting a quite different qualitative–quantitative profile. The antioxidant properties of the extracts were explored through in vitro methods: 1,1-diphenyl-2-picrylhydrazyl (DPPH), reducing power and Fe^2+^ chelating activity assays. The results of the antioxidant tests showed that the extracts possess a different antioxidant ability: particularly, the extract of *M. fruticulosa* subsp. *fruticulosa* exhibited higher radical scavenging activity than that of subsp. *coronopifolia* (IC_50_ = 1.25 ± 0.02 mg/mL and 2.86 ± 0.05 mg/mL), which in turn displayed better chelating properties (IC_50_ = 1.49 ± 0.01 mg/mL and 0.63 ± 0.01 mg/mL). Lastly, *Artemia salina* lethality bioassay was performed for toxicity assessment. The results of the bioassay showed lack of toxicity against brine shrimp larvae for both extracts. The data presented indicate the infraspecific taxa of *M. fruticulosa* as new and safe sources of antioxidant compounds.

## 1. Introduction

*Brassicaceae* plants have been an interesting research topic for years due to their chemical composition characterized by the presence of a variety of bioactive metabolites with valuable potential applications in improving nutrition and human health [1].

In recent years, our research team has focused on the study of taxa that grow spontaneously in Sicily (Italy) included in the *Brassicaceae* family, with the aim of discovering new sources of bioactive compounds that could be used in the pharmaceutical, nutraceutical, and cosmetic fields. In a previous article, we reported the characterization of the phenolic and volatile components and the in vitro antioxidant properties of the aerial part hydroalcoholic extract of *M. incana* subsp. *incana* (L.) R. Br. [2]. Based on the promising results obtained, it seemed interesting to extend our research to the other specific and infraspecific taxa comprised in the genus *Matthiola* R. Br. endemic to Sicily. In particular, the infraspecific taxa of *Matthiola fruticulosa* (L.) Maire were selected for the current study.

*Matthiola fruticulosa* (L.) Maire (synonyms: *Cheiranthus fruticulosus* L., *Matthiola tristis* (L.) R. Br.) is a perennial species that reaches up to 60 cm in height, sparsely pubescent to densely white tomentose, woody at the base. The leaves are linear or oblong, entire to sinuate-pinnatifid, and the flowers are gathered in terminal racemes with yellowish to purplish-violet petals. The fruit is a siliqua, erect or patent, more or less cylindrical [3,4,5].

*Matthiola fruticulosa* is native to Southern Europe (Southeastern Europe: Albania, Bulgaria, Croatia, Greece, Italy, Macedonia, Montenegro, Serbia; Southwestern Europe: France, Portugal, Spain), Northern Africa (Algeria, Libya, Morocco, Tunisia), and Western Asia (Cyprus, Turkey) [3,5,6].

In the “Flora Europaea” and “Flora Hellenica”, as well as in the consulted taxonomic database “The Plant List”, three different subspecies are indicated under *M. fruticulosa*: the nominal subspecies (i.e., subsp. *fruticulosa*), subsp. *valesiaca* (Gay ex Gaudin) P. W. Ball, and subsp. *perennis* (P. Conti) P. W. Ball [5,7,8]. On the other hand, in the latest edition of the “Flora d’Italia” only two infraspecific subdivisions are recognized for this taxon: the nominal subspecies, which prefers calcareous grounds, and the subsp. *coronopifolia* (Sm.) Giardina and Raimondo (synonyms: *Cheiranthus coronopifolius* Sm., *M. tristis* var. *montana* Lojac., *M. tristis* var. *bicornis* Lojac., *M. fruticulosa* var. *tricornis* Lojac.), which prefers clayey and marly substrates [3,9]. Both subspecies occur in Sicily, as reported in the “Checklist of the vascular flora of Sicily” by Raimondo et al. [4].

The use of *M. fruticulosa* as a folk remedy has been documented: actually, its utilization in traditional medicine in Libya for the treatment of kidney stones and piles has been reported [10]. Despite this, as far as we know, in the literature there are no bibliographic data about the phytochemical composition and the evaluation of the biological properties of this taxon, except for the not very recent characterization of a few glucosinolates in the seeds and aerial parts of *M. fruticulosa* collected in Spain [11].

These premises prompted us to design a study aimed at defining the phytochemical profile and the antioxidant properties of the hydroalcoholic extracts obtained from the aerial parts of both *M. fruticulosa* infraspecific taxa growing wild in Sicily, namely, subsp. *fruticulosa* and subsp. *coronopifolia*. A comprehensive insight into the qualitative–quantitative profile of the phenolic and the volatile constituents contained in the extracts was achieved by HPLC–PDA/ESI–MS and SPME–GC/MS analyses. Three different in vitro methods, which are based on different mechanisms, were used to assess the antioxidant activity. Lastly, the brine shrimp (*Artemia salina* Leach) lethality bioassay was utilized to evaluate the toxicity of the extracts.

Both infraspecific taxa of *M. fruticulosa* were found to be safe sources of bioactive compounds with antioxidant properties significantly different from a phytochemical point of view.

## 2. Results and Discussion

### 2.1. Phytochemical Investigations

#### 2.1.1. Determination of Polyphenolic Compounds by HPLC–PDA/ESI–MS

**Figure 1:**
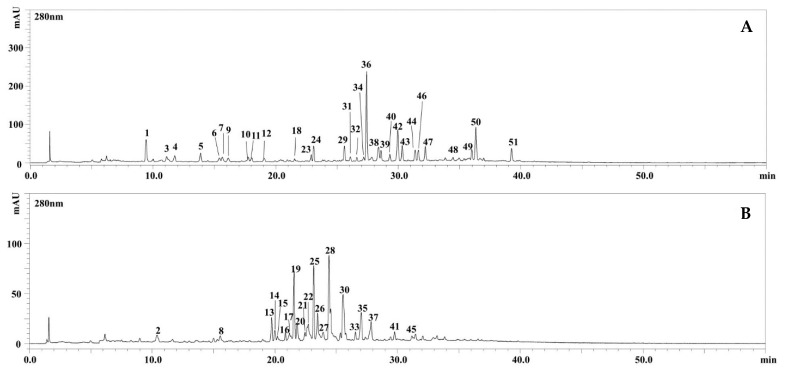
HPLC–PDA chromatograms of the polyphenolic compounds, extracted at 280 nm wavelength, of the hydroalcoholic extracts obtained from the aerial parts of *M. fruticulosa* subsp. *fruticulosa* (**A**) and subsp. *coronopifolia* (**B**). For peak identification, see Table 1.

The polyphenolic characterization of the hydroalcoholic extracts of the aerial parts of the two subspecies of *M. fruticulosa* is reported here for the first time. In Figure 1, the HPLC–PDA chromatograms (λ = 280 nm) of the polyphenolic compounds occurring in the extracts of *M. fruticulosa* subsp. *fruticulosa* (A) and subsp. *coronopifolia* (B) are shown. A total of 51 compounds were detected, 31 in *M. fruticulosa* subsp. *fruticulosa* and 22 in subsp. *coronopifolia* (Table 1). Among them, 20 phenolic compounds were tentatively identified in *M. fruticulosa* subsp. *fruticulosa* according to retention times, PDA, MS, and literature data, 11 belonging to a class of flavonoids and 9 to phenolic acids; on the other hand, 11 compounds were characterized in *M. fruticulosa* subsp. *coronopifolia*, 6 flavonoids, and 5 phenolic acids. The identified flavonoid compounds belong to a class of flavonols, namely, quercetin, kaempferol, and isorhamnetin, and of flavones, namely, luteolin and its derivative isoorientin, while the phenolic acids are benzoic acids, hydroxybenzoic acid and syringic acid, and cinnamic acids, sinapic acid and ferulic acid. Except for peak n. 6 in *M. fruticulosa* subsp. *fruticulosa,* the rest of the polyphenolic compounds occurred in a glycosylated form.

In addition to polyphenols, 4-(methylthio)but-3-enyl glucosinolate (glucoraphenin) and its isomer (peaks n. 3 and n. 4) were detected in traces in the extract of *M. fruticulosa* subsp. *fruticulosa* only. The presence of this glucosinolate compound in a 70% methanol extract of *M. fruticulosa* aerial parts collected in Spain was previously reported by Gmelin and Kjær [12].

Interestingly, from the comparison of the phenolics detected in the two subspecies, a quite different profile was highlighted since no compounds in common were found.

Regarding quantitative determination, the total amount of polyphenols identified in the extract of *M. fruticulosa* subsp. *fruticulosa* was found to be approximately three fold higher than that of subsp. *coronopifolia* (151.6 mg/g extract and 51.8 mg/g extract, respectively), with the flavonoids being 91.7 mg/g extract and 33.5 mg/g extract and the phenolic acids 59.9 mg/g extract and 18.3 mg/g extract. In addition, peak n. 36, feruloylhydroxyferuloyl-dihexoside, was the most abundant polyphenolic compound in *M. fruticulosa* subsp. *fruticulosa* (26.74 mg/g ± 2.48), whereas in *M. fruticulosa* subsp. *coronopifolia,* peak n. 30, kaempferol derivative, turned out to be the main component (11.93 mg/g ± 0.82).

Comparing the polyphenolic profiles of the two subspecies here analyzed with that of the aerial part extract of *M. incana* subsp. *incana* previously investigated, substantial quali-quantitative differences were highlighted [2]. Indeed, the total amount of polyphenols detected in the *M. incana* subsp. *incana* extract (161.31 mg/g extract) was quite similar to that of *M. fruticulosa* subsp. *fruticulosa* and about three times higher than that of subsp. *coronopifolia*, with the flavonoids being the most representative group (155.85 mg/g extract), while the two phenolic acids identified (i.e., vanillic and sinapic acids) occurred in small amounts. On the other hand, phenolic acids were contained in greater numbers in the extracts from the two subspecies of *M. fruticulosa*, accounting their amounts to be about one-third of the total phenols quantified for both of them.

#### 2.1.2. Identification of Volatile Compounds by SPME–GC/MS

Table 2 reports the volatile profile of the hydroalcoholic extracts obtained from the aerial parts of *M. fruticulosa* subsp. * fruticulosa* and subsp. *coronopifolia*. As can be observed in the table, the 61 volatiles identified can be classified into several main groups: aldehydes (mainly with alkanals, 2-alkenals, and 2,4-alcadienals, although also aromatic), acids, alcohols (mainly unsaturated and branched), ketones, esters (mainly methyl esters), sulfides, nitriles, and terpenoids.

As regards *M. fruticulosa* subsp. *fruticulosa,* the extract of the aerial parts was constituted mainly of sulfides (primarily dimethyl trisulfide and dimethyl tetrasulfide), which accounted for more than 77% of all volatiles. Among the other chemical classes, esters (8.09%), aldehydes (5.73%), and ketones (2.78%) were the most represented, with methyl benzoate, nonanal, and 4-methyl-3-penten-2-one as the main compounds in the three classes, respectively. These five volatiles, namely, dimethyl trisulfide, dimethyl tetrasulfide, methyl benzoate, nonanal, and 4-methyl-3-penten-2-one, accounted for more than 88% of the volatile fraction of the *M. fruticulosa* subsp. *fruticulosa* extract.

The extract of the *M. fruticulosa* subsp. *coronopifolia* aerial parts showed a volatile profile in which ketones (31.54%) and terpenes (23.15%) prevailed, although the other classes were also well represented, ranging between 3.17% (acids) and 11.58% (alcohols). The main volatile compounds were 2-isopropyl-5-methyl-3-cyclohexen-1-one among terpenoids; hexahydrofarnesyl acetone, 4-methyl-3-penten-2-one, and 4-methyl-2-pentanone among ketones; 1-octen-3-ol among alcohols; 4-(methylthio)-butanenitrile and 4-methylpentanenitrile among nitriles; 2-methylbutanal among aldehydes; ethyl decanoate among esters; and dimethyl disulfide and octanoic acid among sulfides and acids, respectively.

**Figure 2:**
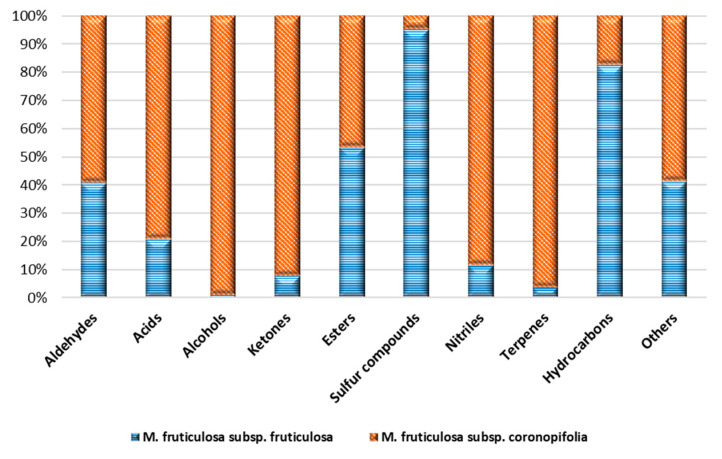
Normalized percentage composition, as classes of substances, of the volatile profile of *M. fruticulosa* subsp. *fruticulosa* and subsp. *coronopifolia* extracts.

Figure 2 shows the normalized percentage composition, as classes of substances, of the two subspecies of *M. fruticulosa*, highlighting considerable differences between them.

In plants, several biosynthetic pathways occur, leading to a wide range of volatile organic compounds as secondary metabolites; thus, differences in the volatile profiles of different plants may reflect differences in their metabolic pathways. These differences have been observed in plants of the same species and even in different cultivars of the same subspecies [13]. The volatile compounds identified in the samples of *M. fruticulosa* subsp. *fruticulosa* and subsp. *coronopifolia* can be classified into terpenoids, carotenoid derivatives, fatty acid derivatives, amino acid derivatives, and benzenoid compounds.

The terpenoids safranal, β-cyclocitral, isophorone, and geranylacetone derive from the enzymatic degradation of carotenoids; the first two compounds were present in both subspecies, whereas the others only in subsp. *coronopifolia*, in any case at a low percentage. The other terpenoid compounds were detected only in the extract of *M. fruticulosa* subsp. *coronopifolia*, and all of them were oxygenated monoterpenes; these compounds have not been reported previously among volatiles of *Matthiola* species. Terpenoids in plants originate from two distinct metabolic pathways: the cytosolic mevalonic acid pathway (MVA) and the plastidial methylerythritol phosphate pathway (MEP). Isopentenyl pyrophosphate (IPP) and dimethylallyl pyrophosphate (DMAPP) are the end products in both pathways and are the precursors of a very large number of compounds by numerous enzyme-catalyzed reactions, including cyclization, hydroxylation, dehydrogenation, oxidation and/or reduction, and isomerization.

Aldehydes, alcohols, and esters, containing 6 to 10 carbon atoms, originate from unsaturated fatty acids through the lipoxygenase pathway. In particular, linoleic and linolenic acid are the precursors of the so-called green leaf volatiles, namely, C6 aldehydes, alcohols, and their ester. Linoleic acid is responsible also for the formation of 1-octen-3-one and 1-octen-3-ol, whereas nonanal, 1-nonanol, heptanal, and 1-heptanol are formed from oleic and palmitoleic acids. Among fatty acid derivative compounds, nonanal was the main volatile in subsp. *fruticulosa*, whereas 1-octen-3-ol was the most abundant in subsp. *coronopifolia*, highlighting possible differences between the two subspecies in both their fatty acid composition and the set of lipoxygenases.

Nitriles are among the amino-acid-derivative compounds. In fact, amino acids are the precursors of glucosinolates, a group of sulfur-containing secondary metabolites characterizing *Brassicaceae* vegetables; from the glucosinolate hydrolysis, through the action of the myrosinase enzyme, isothiocyanates, nitriles, and thiocyanates may arise. In both subspecies of *M. fruticulosa*, only nitriles were detected, with a higher percentage in subsp. *coronopifolia*, where 4-(methylthio)-butanenitrile was the most abundant compound, followed by 4-methylpentanenitrile. 4-(Methylthio)-butanenitrile originates from glucoerucin, which has been identified as the main glucosinolate in *M. fruticulosa* seeds [11]. The absence of isothiocyanates suggests that in our samples the hydrolysis of glucosinolates is altered in favor of nitriles; many factors can modify the activity of myrosinase, favoring nitrile formation [14], such as the presence of ferrous ions, nitrile-specific proteins, acidic conditions, and, according to Wieczorek and Jelen [13], also frozen–thawed processes of the vegetable tissue.

The only sulfur compounds detected in the extracts of *M. fruticulosa* subsp. *fruticulosa* and subsp. *coronopifolia* were sulfides (i.e., dimethyl disulfide, dimethyl trisulfide, and dimethyl tetrasulfide). Sulfides are derivative compounds of the S-alk(en)yl-l-cysteine pathway and occur very frequently in the volatile fraction of *Brassicaceae* family plants, even being the most abundant volatiles in *M. incana* subsp. *incana* and *Brassica incana* grown wild in Sicily [2,15] and in different cultivars of *Brassica oleracea* [13].

Benzenoid compounds are derived from t-cinnamic acid with the propyl side chain shortened by two carbon atoms via either a non-β-oxidative or a β-oxidative pathway, and the formation of benzaldehyde and benzoyl-CoA as intermediates for benzoic acid production [16]. Benzaldehyde and benzoic acid were detected only in the *M. fruticulosa* subsp. *fruticulosa* extract; moreover, methyl benzoate was also present, being the most abundant compound among esters.

Finally, hexahydrofarnesyl acetone was among the most abundant volatiles of the *M. fruticulosa* subsp. *coronopifolia* extract; hexahydrofarnesyl acetone, or phytone, is a ketone very common in plants that arises from the oxidative degradation of (*E*)-phytol, a diterpene alcohol that occurs as a side chain of chlorophyll a [17].

### 2.2. Antioxidant Activity

In the last decades, oxidative stress has been recognized to play a critical role in the pathogenesis of several diseases. This has significantly increased research studies aimed at discovering new plant sources of antioxidants. The *Brassicaceae* family encloses many plant species that are potential sources of antioxidant compounds, including some belonging to the *Matthiola* genus, as also demonstrated in recent studies on *M. incana* infraspecific taxa carried out by our research team [2,18,19,20].

In this work, the antioxidant properties of the extracts of *M. fruticulosa* subsp. *fruticulosa* and subsp. *coronopifolia* were established using three in vitro tests to evaluate the different mechanisms through which the diverse antioxidant compounds contained in the phytocomplexes could exert their effect: DPPH assay, based on the hydrogen atom transfer (HAT) and electron transfer (ET) mechanisms; reducing power, an electron transfer (ET)-based assay; and ferrous ion (Fe^2+^) chelating activity assay.

**Figure 3:**
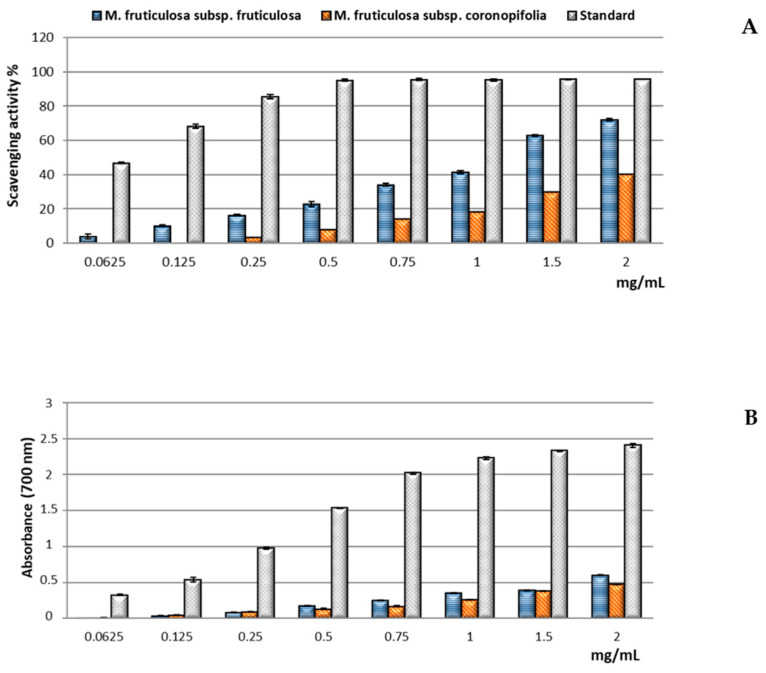

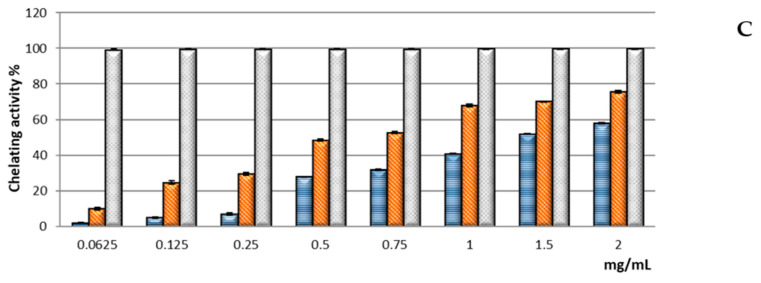
Free radical scavenging activity (DPPH assay) (**A**), reducing power (**B**), and ferrous ion chelating activity (**C**) of the hydroalcoholic extracts obtained from the aerial parts of *M. fruticulosa* subsp. *fruticulosa* and subsp. *coronopifolia*. Reference standard: BHT (**A**,**B**), EDTA (**C**). Values are expressed as the mean ± SD (*n* = 3).

Figure 3A shows the results of the DPPH assay, utilized to determine the free scavenging ability of the extracts. Compared with the reference standard BHT, both extracts displayed lower activity in the range of concentrations tested, which increased with increasing dose. The extract of *M. fruticulosa* subsp. *fruticulosa* exhibited the best scavenging activity, reaching about 70% inhibition of the DPPH radical at the highest concentration tested. From the comparison of the calculated IC_50_ values, it was evidenced that the radical scavenging ability of the *M. fruticulosa* subsp. *fruticulosa* extract, showing an IC_50_ of 1.25 ± 0.02 mg/mL, was approximately 2.3-fold higher than that of the subsp. *coronopifolia* extract (IC_50_ = 2.86 ± 0.05 mg/mL). Comparing the free radical scavenging activity of the *M. fruticulosa* subsp. *fruticulosa* extract with that highlighted in other *Matthiola* species, the result showed an activity higher than that of the hydroalcoholic extracts (80% MeOH) of the aerial parts from the *Matthiola incana* infraspecific taxa endemic to Sicily, whose antioxidant properties had previously been investigated under the same experimental conditions, namely, *M. incana* subsp. *incana*, IC_50_ = 2.32 ± 0.24 mg/mL; subsp. *rupestris*, IC_50_ = 1.73 ± 0.02 mg/mL and 2.60 ± 0.01 mg/mL; and subsp. *pulchella*, IC_50_ = 3.69 ± 0.14 mg/mL [2,18]. On the other hand, Abdelshafeek et al. [20] reported a lower IC_50_ value (0.49 mg/mL) for a hydroalcoholic extract (80% MeOH) obtained by maceration from the aerial parts of *M. longipetala* subsp. *longipetala* collected in Libya. The results of the evaluation of the reducing power of the extracts of the two subspecies of *M. fruticulosa*, determined through the Fe^3+^–Fe^2+^ transformation method, showed that both extracts displayed mild activity compared with the standard BHT. This result agrees with those previously reported for the extracts of the aerial parts from the *M. incana* infraspecific taxa [2,18]. As shown in Figure 3B, the extracts exhibited similar reducing power, dose dependent, although the calculated ASE/mL values indicated greater reducing efficacy for the extract of subsp. *coronopifolia* (18.61 ± 0.06 mg/mL) than that of subsp. *fruticulosa* (38.17 ± 1.14 mg/mL). Both extracts are less active than that of *M. incana* subsp. *incana* (ASE/mL = 12.28 ± 0.42 mg/mL), which showed the best reducing power among the infraspecific taxa of *M. incana* [2,18].

In the Fe^2+^ chelating activity assay, performed by evaluating the inhibiting effect on the Fe^2+^–ferrozine complex formation, both extracts of the two subspecies of *M. fruticulosa* showed good chelating properties, dose dependent, although lower than those of the reference standard EDTA (Figure 3C). Contrary to what has been observed in the other antioxidant assays, the extract of *M. fruticulosa* subsp. *coronopifolia* displayed the best chelating activity, reaching about 75% inhibition at the highest concentration tested. This was confirmed also by a comparison of the IC_50_ values calculated for the extracts, which indicated that the chelating efficacy of *M. fruticulosa* subsp. *coronopifolia* (0.63 ± 0.01 mg/mL) was approximately 2.5-fold higher than that of *M. fruticulosa* subsp. *fruticulosa* (1.49 ± 0.01 mg/mL). The chelating activity of the extract of *M. fruticulosa* subsp. *coronopifolia* was close to that previously found for that of *M. incana* subsp. *incana* (IC_50_ = 0.53 ± 0.02 mg/mL), the most effective among those from the infraspecific taxa of *M. incana* [2,18].

The results of the antioxidant tests clearly indicate that the extracts of the two subspecies of *M. fruticulosa* possess different antioxidant ability; in fact, the extract of *M. fruticulosa* subsp. *fruticulosa* exhibited higher radical scavenging activity than that of subsp. *coronopifolia*; conversely, the latter displayed better chelating properties than the former.

Among plant secondary metabolites, polyphenols represent the most important group of antioxidant compounds. Flavonoids and phenolic acids, the largest classes of plant phenolics, have been shown to be effective antioxidants in several in vitro and in vivo investigations [21,22,23]. The higher radical scavenging properties of the extract of *M. fruticulosa* subsp. *fruticulosa* seem to be related to the greater content of flavonoids and phenolic acids detected by HPLC–PDA/ESI–MS analysis, both present in quantities about three times higher than those of subsp. *coronopifolia*. Regarding the chelating properties, which are greater for *M. fruticulosa* subsp. *coronopifolia*, although the partial involvement of the phenolic constituents detected in the extracts cannot be ruled out, it is assumed that they may depend on other antioxidant phytochemicals.

### 2.3. Artemia salina Leach Lethality Bioassay

*Artemiasalina* Leach (brine shrimp) is a small crustacean widely utilized as a model organism in the toxicity assessment of plant extracts [24]. The brine shrimp lethality bioassay has been applied as an alternative method for the preliminary estimation of toxicity because it shows several advantages, such as rapidity, cost-effectiveness, ease of handling and maintenance under laboratory conditions, and adaptability to various testing conditions [25].

The results of the bioassay showed the absence of toxicity against brine shrimp larvae for both extracts of the *M. fruticulosa* subspecies. Indeed, the median lethal concentration values were found to be above 1000 μg/mL, thus indicating their potential safety based on Clarkson’s toxicity criterion [26]. The obtained results agree with those reported for the hydroalcoholic extracts of the aerial parts from the *M. incana* infraspecific taxa previously investigated, which were tested under the same experimental conditions [2,18].

## 3. Materials and Methods

### 3.1. Chemicals and Reagents

LC–MS-grade water (H_2_O), acetonitrile (ACN), gallic acid, catechin, chlorogenic acid, apigenin, luteolin, rutin, kaempferol, and quercetin were obtained from Merck Life Science (Merck KGaA, Darmstadt, Germany). LC–MS-grade formic acid was purchased from Riedel-de Haën (Seelze, Germany). Methanol (MeOH) was purchased from Carlo Erba (Milan, Italy). Unless indicated otherwise, all chemicals were purchased from Sigma-Aldrich (Milan, Italy).

### 3.2. Plant Material and Extraction Procedure

The aerial parts of the *Matthiola fruticulosa* subspecies were collected in Sicily (Italy): *M. fruticulosa* subsp. *fruticulosa* (L.) Maire was picked in May 2019 in the locality of Polizzi Generosa, Contrada Pietà (Palermo), on a dolomitic lithosol, about 1200 m (a.s.l., above sea level), and *M. fruticulosa* subsp. *coronopifolia* (Sm.) Giardina and Raimondo in the locality of Sutera (Caltanissetta), on chalky cliffs, about 400 m (a.s.l.), in June 2019. Voucher specimens were identified by Prof. F.M. Raimondo, PLANTA/Center for Research, Documentation and Training (Palermo), and Prof. V. Spadaro, University of Palermo, and have been deposited to the Herbarium Mediterraneum of the University of Palermo, Italy (PAL-Gr) (voucher numbers: *Raimondo & Spadaro* n. 03/19; *Schimmenti* n. 05/19).

After harvesting, the aerial parts of the two subspecies were immediately frozen; then after lyophilization, the plant material was subjected to a preventive maceration with 80% MeOH (1:10 *w*/*v*) for 150 min. The extraction was performed with 80% MeOH (1:10 *w*/*v*) in an ultrasonic bath at 50 °C for 15 min three times; then the filtrates were pooled and evaporated to dryness by a rotavapor. The yields of the extracts, referring to 100 g of lyophilized plant material, were 20.38% and 19.83% for *M. fruticulosa* subsp. *fruticulosa* and subsp. *coronopifolia*, respectively.

### 3.3. Phytochemical Investigations

#### 3.3.1. Identification of Phenolic Compounds by HPLC–PDA/ESI–MS

The analyses were carried out using a Shimadzu HPLC system (Milan, Italy) equipped with a CBM-20A controller, LC-20AD pumps, a DGU-20A3 degasser_,_ a SIL-20AC autosampler, an SPD-M20A photodiode array detector (PDA), and a triple quadrupole mass analyzer (LCMS-8050, Shimadzu, Kyoto, Japan), equipped with an ESI interface, in positive and negative ionization modes. Data acquisition was performed by Shimadzu LabSolutions software ver. 5.91.

*Samples and sample preparation*: An amount of 20 mg of *M. fruticulosa* subsp. *fruticulosa* and subsp. *coronopifolia* extracts was dissolved in 1 mL of MeOH.

*Chromatographic conditions:* Analyses were carried out on an Ascentis Express C18, 15 cm × 4.6 mm I.D. with a particle size of 2.7 µm (Merck Life Science, Merck KGaA, Darmstadt, Germany). The injection volume was 5 µL, the mobile phase consisted of water/formic acid (99.9:0.1, *v*/*v*) (solvent A) and ACN/formic acid (99.9:0.1, *v*/*v*) (solvent B), and the linear gradient profile was as follows: 0 min, 0% B; 5 min, 5% B; 15 min, 10% B; 30 min, 20% B; 60 min, 50% B; 70 min, 100% B; 71 min, 0% B. The flow rate was 1 mL/min, and it was split to 0.2 mL/min prior to MS detection.

*PDA conditions:* The wavelength range was 200–400 nm, and the chromatograms were extracted at a wavelength of 280 nm. Time constant was 0.08 s, and the sample frequency was 40 Hz.

*MS conditions:* The mass spectral range was 100–1000 *m*/*z*, the interval was 0.5 s, the nebulizing gas (N_2_) flow was 1.5 L/min, the interface temperature was 350 °C, the heat block was 300 °C, the DL temperature was 300 °C, the DL voltage was −34 V, the probe voltage was 4.5 kV, the Qarray voltage was 1.0 V, the RF voltage was 90 V, and the detection gain was 1.0 kV.

Quantitative determination was performed using the calibration curves of six standards, namely, gallic acid, hydroxybenzoic acid, chlorogenic acid, luteolin, kaempferol, and quercetin. Standard calibration curves were prepared in the concentration range of 0.1–50 mg/L with five different concentration levels [27].

#### 3.3.2. Identification of Volatile Compounds by SPME–GC/MS

*Extraction (HS–SPME)*: The hydroalcoholic extracts of the aerial parts of *M. fruticulosa* subsp. *fruticulosa* and subsp. *coronopifolia* were analyzed for their volatile composition by HS–SPME–GC/MS.

An amount of 30.0 mg of each dried extract was solubilized in 3.0 mL of saturated sodium chloride solution (final concentration of 10 mg/mL) into a 7 mL vial, then closed with a “mininert” valve (Supelco, Bellefonte, PA, USA). The samples were extracted using a DVB/CAR/PDMS fiber with a 50/30 μm film thickness (Supelco, Bellefonte, PA, USA) following the method reported by Miceli et al. [2].

*Analysis (GC/MS)*: The volatiles were analyzed by a Shimadzu GC 2010 Plus gas chromatograph coupled to a TQMS 8040 triple quadrupole mass spectrometer (Shimadzu, Milan, Italy) on two different capillary columns: (1) VF-WAXms, 60 m, 0.25 mm i.d., 0.25 μm film thickness polar column (Agilent Technologies Italia S.p.A., Milan, Italy), and (2) DB-5ms, 30 m, 0.25 mm i.d., 0.25 μm film thickness polar column (Agilent Technologies Italia S.p.A., Milan, Italy).

The conditions were as follows: injection mode, splitless; oven temperature, (1) 45 °C held for 5 min, then increased to 80 °C at a rate of 10 °C/min and to 240 °C at 2 °C/min, held at 240 °C for 5 min, for VF-WAXms column, and (2) 45 °C increased to 160 °C at a rate of 3 °C/min and to 260 °C at 10 °C/min, held at 260 °C for 5 min, for DB-5ms column; carrier gas, helium at a constant flow of 1 mL/min; transfer line temperature, 250 °C; acquisition range, 40 to 360 *m*/*z*; scan speed, 1250. For the identification of the volatiles, mass spectral data, NIST 14 (NIST/EPA/NIH Mass Spectra Library, version 2.0, NIST Mass Spectrometry Data Center, National Institute of Standards and Technology, Gaithersburg, MD, USA) and FFNSC 3.0 databases, linear retention indexes (LRIs), literature data, and injection of the available standards were used [28].

### 3.4. Antioxidant Activity

#### 3.4.1. DPPH Assay

The 1,1-diphenyl-2-picrylhydrazyl (DPPH) assay was used to determine the free radical scavenging activity of the hydroalcoholic extracts of *M. fruticulosa* subsp. *fruticulosa* and subsp. *coronopifolia* [29]. The extracts were tested in the range of 0.0625–2 mg/mL using butylated hydroxytoluene (BHT) as positive control. A 0.5 mL aliquot of each sample solution was added to 3 mL of DPPH methanol solution (0.1 mM). The mixture was left at room temperature in the dark for 20 min, and then absorbance was measured at 517 nm using a model UV-1601 spectrophotometer (Shimadzu). The results were obtained from the average of three independent experiments, and are reported as mean radical scavenging activity (%) ± standard deviation (SD) and mean 50% inhibitory concentration (IC_50_) ± SD.

#### 3.4.2. Reducing Power Assay

The reducing power of the hydroalcoholic extracts of *M. fruticulosa* subsp. *fruticulosa* and subsp. *coronopifolia* was assessed by the Fe^3+^–Fe^2+^ transformation method [30]. The extracts were tested in the range of 0.0625–2 mg/mL using butylated hydroxytoluene (BHT) and ascorbic acid as positive controls. An amount of 1 mL of each sample solution was mixed with 2.5 mL of phosphate buffer (0.2 M, pH 6.6) and 2.5 mL of 1% potassium ferricyanide. After incubation at 50 °C for 20 min and rapid cooling, 2.5 mL of 10% trichloroacetic acid was added. Finally, 2.5 mL of the supernatant was mixed with 2.5 mL of distilled water and 0.5 mL of 0.1% ferric chloride; then the mixture was incubated for 10 min at room temperature in the dark, and the optical density change was measured at 700 nm. The results were obtained from the average of three independent experiments, and are expressed as mean absorbance values ± SD and ascorbic acid equivalent/mL (ASE/mL) ± SD.

#### 3.4.3. Ferrous Ion (Fe^2+^) Chelating Activity Assay

The spectrophotometric measurement of the Fe^2+^–ferrozine complex was used to determine the Fe^2+^ chelating activity of the hydroalcoholic extracts of *M. fruticulosa* subsp. *fruticulosa* and subsp. *coronopifolia* according to the method previously reported by Decker and Welch [31]. The extracts were tested in the range of 0.0625–2 mg/mL using ethylenediaminetetraacetic acid (EDTA) as positive control. Briefly, 0.05 mL of 2 mM ferrous chloride was added to 1 mL of sample solution and 0.5 mL of methanol. The reaction was initiated by the addition of 0.2 mL of 5 mM ferrozine solution. After vigorous shaking, the mixture was incubated at room temperature in the dark for 10 min; then the optical density change was measured spectrophotometrically at 562 nm. The results were obtained from the average of three independent experiments and are reported as mean inhibition of the ferrozine–(Fe^2+^) complex formation (%) ± SD and IC_50_ ± SD.

### 3.5. Artemia salina Leach Lethality Bioassay

To establish the acute toxicity of the hydroalcoholic extracts of *M. fruticulosa* subsp. *fruticulosa* and subsp. *coronopifolia*, the brine shrimp (*Artemia salina* Leach) lethality bioassay was carried out according to the method of Meyer et al. [32]. Brine shrimp eggs were placed in a brine shrimp hatchery dish filled with sterile artificial seawater for 48 h. After hatching, active nauplii free from eggshells were collected from the brighter portion of the hatchery dish and used for the assay. Ten brine shrimp larvae were incubated for 24 h at 25–28 °C in 5 mL of artificial seawater mixed with different amounts of the extracts (10–1000 µg/mL). Then, the surviving larvae were counted using a magnifying glass, and median lethal concentration (LC_50_) values were determined by Litchfield and Wilcoxon’s method. The assay was carried out in triplicate. The toxicity level of the extracts was assessed according to the toxicity scale reported by Clarkson et al. [26]; extracts giving LC_50_ values greater than 1000 μg/mL were considered nontoxic.

## 4. Conclusions

In summary, in this work we explored for the first time the phytochemical profile and the antioxidant properties of the hydroalcoholic extracts of the aerial parts of *M. fruticulosa* subsp. *fruticulosa* and subsp. *coronopifolia* endemic to Sicily (Italy). From the comparison of the extracts of the two infraspecific taxa of *M. fruticulosa*, a quite different qualitative–quantitative profile of both phenolic and volatile compounds was highlighted, as well as a diverse antioxidant ability. It is interesting to note, in fact, that the extract of *M. fruticulosa* subsp. *fruticulosa* exhibited much higher radical scavenging activity, while that of subsp. *coronopifolia* was shown to be a better source of metal chelating antioxidants. Furthermore, the lack of toxicity against brine shrimp larvae indicates the potential safety of the extracts.

Overall, the data presented here improve the knowledge of the taxa included in the *Matthiola* genus, also indicating the infraspecific taxa of *M. fruticulosa* endemic to Sicily as new and safe sources of bioactive compounds, which increases the number of *Brassicaceae* plants hitherto known for their health-promoting properties. The present findings pave the way for new studies to further investigate the antioxidant properties of these taxa and to evaluate their potential protective effect against diseases related to oxidative stress.

## Figures and Tables

**Table 1:** HPLC–PDA/ESI–MS (negative and positive ionization modes) polyphenolic fingerprint of *M. fruticulosa* subsp. *fruticulosa* (**A**) and subsp. *coronopifolia* (**B**) extracts. Results are expressed as mg/g extract ± S.D. (*n* = 3).

N^o^	t_R_ (min)	UV_max_ (nm)	[M − H]^−^	[M + H]^+^	*Tentative* *Identification*	mg/g Extract	
						A	B
**1**	9.3	260	299	-	Hydroxybenzoic acid-hexoside	6.21 ± 1.73	n.d.
**2**	10.4	276	359	-	Syringic acid-hexoside	n.d.	1.64 ± 0.03
**3**	11.0	264, 297	418	-	4-(methylthio)but-3-enyl glucosinolate	n.q.	n.d.
**4**	11.7	293	418	-	4-(methylthio)but-3-enyl glucosinolate isomer	n.q.	n.d.
**5**	13.8	257, 295	387	-	Unknown	n.q.	n.d.
**6**	15.3	277	325	-	5-hydroxyferuloylmalate	1.71 ± 0.30	n.d.
**7**	15.5	315	289	-	Unknown	n.q.	n.d.
**8**	15.6	308	413	-	Unknown	n.d.	n.q.
**9**	16.0	232	329	-	Unknown	n.q.	n.d.
**10**	17.6	330	517	-	Feruloyl-dihexoside	3.01 ± 0.67	n.d.
**11**	17.9	329	355	-	Ferulic acid-dihexoside	3.23 ± 0.78	n.d.
**12**	19.0	330	385	-	Sinapoylhexoside	3.91 ± 0.72	n.d.
**13**	19.7	269_sh_, 329	437	-	Unknown	n.d.	n.q.
**14**	20.0	267_sh_, 314	725,433,285	595	Kaempferol derivative	n.d.	1.82 ± 0.01
**15**	20.2	267_sh_, 328	411	-	Unknown	n.d.	n.q.
**16**	20.9	268_sh_, 329	423,379	-	Unknown	n.d.	n.q.
**17**	21.2	337	371	-	Hydroxyferuloyl-hexoside	n.d.	1.62 ± 0.07
**18**	21.4	349	755,609,285	449	Kaempferol derivative	1.93 ± 0.36	n.d.
**19**	21.6	269_sh_, 328	595,440,285	339	Kaempferol-dihexoside	n.d.	10.99 ± 0.26
**20**	21.8	269_sh_, 318	739,579,285	-	Kaempferol-trihexoside	n.d.	3.23 ± 0.15
**21**	22.5	329	739,579	595	Sinapoylhydroxyferuloyl-dihexoside	n.d.	1.53 ± 0.18
**22**	22.6	349	741,301	-	Quercetin derivative	n.d.	3.03 ± 0.37
**23**	22.8	266, 337	185	-	Unknown	n.q.	n.d.
**24**	23.0	329	739,579	595	Sinapoylhydroxyferuloyl-dihexoside isomer	4.93 ± 1.25	n.d.
**25**	23.2	268_sh_, 336	387,501	438	Unknown	n.d.	n.q.
**26**	23.5	269_sh_, 314	387	-	Unknown	n.d.	n.q.
**27**	23.9	311	251	-	Unknown	n.d.	n.q.
**28**	24.4	266, 349	725	433	Dihydroxyferuloyl-hexoside	n.d.	10.83 ± 0.04
**29**	25.5	255, 350	725	433	Dihydroxyferuloyl-hexoside isomer	5.63 ± 1.30	n.d.
**30**	25.6	330	755,515,435,285	-	Kaempferol derivative	n.d.	11.93 ± 0.82
**31**	26.0	353	579,303,285	449	Kaempferol-trihexoside	2.74 ± 1.79	n.d.
**32**	26.5	266, 346	458	-	Unknown	n.q.	n.d.
**33**	26.6	338	593,447	-	Isoorientin-hexoside	n.d.	2.76 ± 0.68
**34**	27.0	352	771,301	303	Quercetin-p-coumaroylhexoside	3.65 ± 0.47	n.d.
**35**	27.0	339	695	433	Unknown	n.d.	n.q.
**36**	27.3	266, 346	709,563,431	287	Feruloylhydroxyferuloyl-dihexoside	26.74 ± 2.48	n.d.
**37**	27.9	318	725,563,593	-	Dihydroxyferuloyl-dihexoside isomer	n.d.	2.77 ± 0.52
**38**	28.2	265, 347	755,609,285	-	Kaempferol derivative	10.12 ± 3.28	n.d.
**39**	28.5	329	739,579	-	Sinapoylhydroxyferuloyl-dihexoside isomer	4.74 ± 0.88	n.d.
**40**	29.2	254, 351	593,285	463,287	Luteolin-dihexoside	3.75 ± 2.36	n.d.
**41**	29.8	329	613,518	-	Unknown	n.d.	n.q.
**42**	29.8	265,348	785,755,593,285	287	Kaempferol-feruloyldihexoside	19.85 ± 3.16	n.d.
**43**	30.2	265,348	785,755,593,285	-	Kaempferol-feruloyldihexoside isomer	10.25 ± 2.86	n.d.
**44**	31.3	265, 345	593,285	287	Kaempferol-dihexoside	6.16 ± 0.85	n.d.
**45**	31.5	329	759,449	-	Unknown	n.d.	n.q.
**46**	31.5	254, 352	623,447,315	479,317	Isorhamnetin-dihexoside	12.96 ± 3.94	n.d.
**47**	32.1	254, 352	623,447,315	479,317	Isorhamnetin-dihexoside isomer	17.63 ± 1.85	n.d.
**48**	34.4	330	447,409,285	181	Kaempferol-hexoside	3.14 ± 0.26	n.d.
**49**	35.9	330	535	495,287	Unknown	n.q.	n.d.
**50**	36.2	330	539	317,287	Unknown	n.q.	n.d.
**51**	39.2	310	674	339	Unknown	n.q.	n.d.

n.d.: not detected; n.q.: not quantified.

**Table 2:** Composition as volatile constituents and classes of substances of the hydroalcoholic extracts obtained from the aerial parts of *M. fruticulosa* subsp. *fruticulosa* and subsp. *coronopifolia*.

Compound	LRI * on DB-5ms	LRI * on VF-AXms	*M. fruticulosa* Subsp. *fruticulosa*		*M. fruticulosa* Subsp. *coronopifolia*	
			Amount ^†^ X ± Dev St	Percentage X ± Dev St	Amount ^†^ X ± Dev St	Percentage X ± Dev St
**Aldehydes**						
2-Methylbutanal	675	913	-^§^	-	1262 ± 143	3.73 ± 0.39
Heptanal	903	1198	-	-	51 ± 7	0.15 ± 0.02
(*E*)-2-Heptenal	957	1338	-	-	396 ± 43	1.17 ± 0.19
Benzaldehyde	962	1529	261 ± 47	0.22 ± 0.02	298 ± 38	0.88 ± 0.11
Octanal	1004	1298	508 ± 51	0.43 ± 0.05	101 ± 19	0.30 ± 0.05
(*E*,*E*)-2,4-Heptadienal	1014	1497	-	-	247 ± 34	0.73 ± 0.14
Phenylacetaldehyde	1043	1640	-	-	213 ± 37	0.63 ± 0.11
Nonanal	1105	1398	5554 ± 939	4.72 ± 0.74	-	-
(*E*)-2-Nonenal	1161	1543	72 ± 12	0.06 ± 0.01	-	-
Decanal	1206	1502	115 ± 19	0.10 ± 0.02	116 ± 14	0.34 ± 0.06
(*E*)-2-Decenal	1263	1647	-	-	101 ± 12	0.30 ± 0.05
(*Z*)-9-Octadecenal	2006	2693	242 ± 42	0.21 ± 0.03	-	-
*All*			*6752 ± 385*	*5.73 ± 0.30*	*2787 ± 55*	*8.24 ± 0.16*
**Acids**						
Benzoic acid	1165	2433	522 ± 60	0.47 ± 0.08	-	-
Octanoic acid	1174	2072	262 ± 29	0.22 ± 0.04	636 ± 96	1.88 ± 0.31
Nonanoic acid	1269	2178	180 ± 26	0.15 ± 0.02	231 ± 30	0.68 ± 0.09
Decanoic acid	1366	2284	-	-	205 ± 36	0.61 ± 0.11
*All*			*995 ± 41*	*0.84 ± 0.05*	*1072 ± 62*	*3.17 ± 0.20*
**Alcohols**						
(*E*)-2-Hepten-1-ol	973	1515	-	-	409 ± 78	1.21 ± 0.19
1-Octen-3-ol	980	1452	-	-	2719 ± 342	8.04 ± 1.09
2-Ethyl-1-hexanol	1029	1489	164 ± 20	0.14 ± 0.02	-	-
(*E*)-2-Octen-1-ol	1068	1617	-	-	244 ± 35	0.72 ± 0.14
1-Octanol	1072	1561	-	-	393 ± 71	1.16 ± 0.21
2-Methyl-1-octanol	1100	-	-	-	148 ± 27	0.44 ± 0.08
*All*			*164 ± 20*	*0.14 ± 0.02*	*3914 ± 161*	*11.58 ± 0.51*
**Ketones**						
4-Methyl-2-pentanone	736	1010	-	-	487 ± 90	1.44 ± 0.23
2-Hexanone	790	1083	-	-	265 ± 36	0.78 ± 0.15
4-Methyl-3-penten-2-one	801	1132	2267 ± 228	1.92 ± 0.39	4097 ± 746	12.12 ± 2.43
Acetylacetone	815	1196	-	-	278 ± 50	0.82 ± 0.14
2-Heptanone	891	1185	-	-	201 ± 29	0.60 ± 0.09
3-Methyl-2-heptanone	936	1210	446 ± 77	0.38 ± 0.07	499 ± 77	1.48 ± 0.19
Acetophenone	1066	1656	249 ± 39	0.21 ± 0.04	-	-
Hexahydrofarnesyl acetone	1844	2121	318 ± 58	0.27 ± 0.04	4835 ± 717	14.3 ± 2.01
*All*			*3279 ± 125*	*2.78 ± 0.20*	*10663 ± 394*	*31.54 ± 1.20*
**Esters**						
Methyl heptanoate	1023	1293	693 ± 136	0.59 ± 0.09	-	-
Methyl benzoate	1094	1628	6239 ± 1218	5.3 ± 1.02	-	-
Methyl octanoate	1123	1394	363 ± 65	0.31 ± 0.05	94 ± 10	0.28 ± 0.05
Ethyl octanoate	1196	1439	126 ± 19	0.11 ± 0.01	135 ± 14	0.40 ± 0.05
Methyl decanoate	1323	1594	216 ± 34	0.18 ± 0.03	64 ± 8	0.19 ± 0.03
Ethyl 9-decenoate	1386	1678	180 ± 24	0.15 ± 0.03	395 ± 66	1.17 ± 0.19
Ethyl decanoate	1394	1639	1078 ± 128	0.92 ± 0.14	1193 ± 216	3.53 ± 0.56
Fumaric acid, pent-4-en-2-yl propyl ester	1492	-	371 ± 55	0.32 ± 0.05	146 ± 18	0.43 ± 0.08
Ethyl dodecanoate	1590	1840	-	-	60 ± 8	0.18 ± 0.04
Methyl tetradecanoate	1726	1997	-	-	40 ± 6	0.12 ± 0.01
Methyl hexadecanoate	1926	2199	264 ± 39	0.22 ± 0.02	192 ± 35	0.57 ± 0.09
Isopropyl hexadecanoate	2023	2232	-	-	53 ± 8	0.16 ± 0.03
*All*			*9530 ± 412*	*8.09 ± 0.35*	*2373 ± 73*	*7.02 ± 0.19*
**Sulfur compounds**						
Dimethyl disulfide	743	1082	855 ± 111	0.73 ± 0.12	855 ± 137	2.53 ± 0.49
Dimethyl trisulfide	968	1391	80953 ± 14196	68.73 ± 13.01	539 ± 97	1.6 ± 0.29
Dimethyl tetrasulfide	1215	1750	9554 ± 1063	8.11 ± 1.29	-	-
*All*			*91362 ± 8219*	*77.57 ± 7.55*	*1395 ± 119*	*4.13 ± 0.40*
**Nitriles**						
4-Methylpentanenitrile	840	1253	-	-	778 ± 110	2.3 ± 0.47
Hexanenitrile	877	1308	-	-	333 ± 61	0.98 ± 0.16
Heptanenitrile	978	1408	855 ± 103	0.73 ± 0.09	-	-
4-(Methylthio)-butanenitrile	1082	1806	-	-	1629 ± 257	4.79 ± 0.85
Benzyl nitrile	1137	1893	409 ± 66	0.35 ± 0.07	-	-
*All*			*1264 ± 87*	*1.08 ± 0.08*	*2730 ± 165*	*8.08 ± 0.57*
**Terpenoids**						
Isophorone	1126	1621	-	-	35 ± 4	0.10 ± 0.01
Cryptone	1175	1675	-	-	289 ± 37	0.85 ± 0.13
Safranal	1200	1649	891 ± 127	0.76 ± 0.11	208 ± 39	0.61 ± 0.13
β-Cyclocitral	1220	1626	246 ± 49	0.21 ± 0.03	179 ± 33	0.53 ± 0.09
Pulegone	1233	1637	-	-	166 ± 22	0.49 ± 0.07
Thymoquinone	1250	-	-	-	171 ± 19	0.50 ± 0.07
2-Isopropyl-5-methyl-3-cyclohexen-1-one	1255	-	-	-	6605 ± 1113	19.54 ± 3.76
Carvacrol	1291	2225	-	-	89 ± 14	0.26 ± 0.04
Geranylacetone	1447	1857	-	-	86 ± 13	0.26 ± 0.05
*All*			*1136 ± 96*	*0.97 ± 0.08*	*7827 ± 372*	*23.15 ± 1.26*
**Hydrocarbons**						
9-Octadecene	1744	1795	554 ± 80	0.47 ± 0.07	-	-
9-Eicosene	1948	1914	280 ± 38	0.24 ± 0.04	50 ± 7	0.15 ± 0.03
*All*			*834 ± 63*	*0.71 ± 0.06*	*50 ± 7*	*0.15 ± 0.03*
**Others**						
*All*			*2463 ± 368*	*2.09 ± 0.42*	*995 ± 179*	*2.94 ± 0.58*

* Linear retention indexes calculated according to the Van Den Dool and Kratz equation; **^†^** peak area arbitrary scale; ^§^ not detected.

## Data Availability

The data presented in this study are available on request from the corresponding author.

## Sample Availability

Samples of the compounds are not available from the authors.
